# Nonparametric regression estimation using multivariable truncated splines for binary response data

**DOI:** 10.1016/j.mex.2024.103084

**Published:** 2024-12-05

**Authors:** Afiqah Saffa Suriaslan, I Nyoman Budiantara, Vita Ratnasari

**Affiliations:** Department of Statistics, Faculty of Science and Data Analytics, Institut Teknologi Sepuluh Nopember, Kampus ITS- Sukolilo, Surabaya 60111, Indonesia

**Keywords:** Binary response data, Truncated spline, Nonparametric regression

## Abstract

In recent years, Truncated Spline estimators in nonparametric regression for quantitative data have gained significant attention. However, in practical applications, it is common to encounter situations where the response variable is qualitative (binary). As a result, Truncated Spline nonparametric regression models designed for quantitative data cannot be directly applied to binary response cases. Therefore, a method is needed that able to handle the relationship between variables whose patterns change at certain sub-intervals, where the response is binary. This article aims to develop a multivariable Truncated Spline nonparametric regression estimator specifically for binary response data. The proposed method is applied to analyze unmet need achievement status in East Java Province, Indonesia, and the percentage of the poor population in Indonesia. The findings indicate that the Truncated Spline nonparametric regression method provides more accurate estimates compared to binary logistic regression. Some of the highlights of the proposed method are:

• This research develops a nonparametric truncated spline regression model tailored for binary response data analysis.
• Using the Akaike Information Criterion (AIC) to select optimal knot points.
• Evaluating the performance of the proposed model in comparing performance of nonparametric Truncated Spline model for binary response and binary logistic regression with the data real application.

## Graphical abstract

**Figure:**
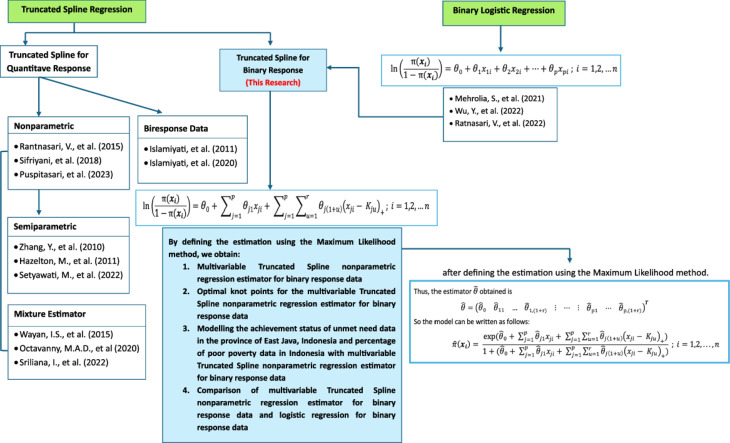

Specifications table

**Table:** 

Subject area:	Mathematics and Statistics
More specific subject area:	Statistics; Nonparametric Regression; Categorical Data
Name of your method:	Truncated Spline; Maximum Likelihood Estimation
Name and reference of original method:	• Spline in the book of Eubank, R.L. (1998), Smoothing and Nonparametric Regression in the. Marcel Dekker, New York. • Maximum likelihood estimation in the book of Hosmer, D. W., & Lemeshow, S. (2000), Applied Logistic Regression, 2nd Edition. United States of America, Canada.
Resource availability:	• Achievement status data of unmet need targets in East Java Province and its predictor variables in 2023 in 38 districts/cities in East Java Province, Indonesia can be accessed on the BKKBN official website (https://portalpk.bkkbn.go.id/tabulasi). • Percentage of poor population and its predictor variables in 2023 in 34 provinces in Indonesia can be accessed on the BPS official website (https://www.bps.go.id/id).

## Background

One of the statistical methods in modeling that is often used in various studies is regression analysis. Modeling in statistics generally pays attention to mathematical and model simplicity, model interpretation, and ease of implementation [1]. One of approach in regression analysis is nonparametric regression. Nonparametric regression has high flexibility properties so that it is well used to determine the pattern of the relationship between response and predictor variables where the function of the regression curve is unknown. The data is expected to find its own form of regression curve estimation without being influenced by the subjectivity of the researcher [2]. Nonparametric regression curves are assumed to be smooth. Nonparametric estimation can be performed based on observed data using smoothing techniques. One of the nonparametric approaches often developed by researchers is Spline. Spline estimators is well used in data cases where the response variable and predictor variables show changing patterns at certain sub-intervals [3].

There are several studies [[4], [5], [6], [7]] that use the Spline nonparametric estimator. Research [[8], [9], [10]] used Truncated Spline estimator in nonparametric regression. Furthermore, [[11], [12], [13]] applied Truncated Spline in semiparametric regression. [14,15] developed biresponse regression using Truncated Spline, until the development of the Truncated Spline nonparametric regression mixture estimator by [[16], [17], [18], [19]]. However, these studies still only discuss cases with quantitative response data, while in reality, there are often cases that have a relationship between response variables and predictor variables where the response is binary. As a result, the Truncated Spline nonparametric regression model that has been developed by previous researchers cannot accommodate cases where the response variable is binary. One of method that can accommodate cases with binary responses is binary logistic regression analysis, where binary logistic regression can be used to explain the relationship between binary response variables and one or more predictor variables. There are several studies that develop binary logistic regression methods [[20], [21], [22]], but these studies have not been able to accommodate cases where the data pattern changes in certain sub-intervals. Whereas in reality, there are cases where the data pattern has a changing pattern at certain sub-intervals. So it is necessary to develop a theory for cases where the data pattern changes in certain sub-intervals and the response variable is binary.

This study aims to review and develop the theory in nonparametric regression modeling, especially Truncated Spline for binary response. The model obtained is then applied to data on the status of achieving the unmet need target in East Java province, Indonesia and to data on the percentage of the poor population in Indonesia. There are 3 predictor variables each to be used. According to the Central Bureau of Statistics (BPS), East Java is one of the provinces with the largest population in Indonesia. Population growth in East Java raises the issue of population density. One aspect that affects the increase in population growth is the birth rate. Therefore, a way to reduce the birth rate is needed [23]. One of the factors causing the high birth rate is the high percentage of unmet need. Representatives of the National Population and Family Planning Agency (BKKBN) stated that unmet need in East Java based on data from the Family Planning Information System (SIGA-YAN) in 2023 was still high, at 12.97 %. Meanwhile, the unmet need target is 11.74 %. On the other hand, the percentage of poor population in Indonesia is a major concern in social and economic policy planning. According to BPS, the percentage of poor population in Indonesia in 2023 reached 9.03 %. This figure shows the challenges for the government in reducing the percentage of poor population to ensure an evenly distributed improvement in population's welfare, as well as to support sustainable economic growth in all regions of Indonesia. Therefore, an appropriate analysis is needed and involves all components of the problem in handling it.

In this study, data preprocessing involved several important steps to ensure the reproducibility of the analysis. First, the response variables are classified into category 1 and category 0, according to national standards relevant to the research context. Secondly, multicollinearity was assessed to ensure the absence of strong interrelationships among the predictor variables. Next, using the likelihood function for the developed truncated spline model with a binary response, the first and second derivatives will be computed to obtain optimal parameter estimates. This procedure is followed by numerical iteration until convergence is achieved, defined as a change in iteration values <0.000001. Data analysis was conducted using R software, which can facilitates model estimation and model performance comparison. This study introduces a novel theoretical framework for the Multivariable Truncated Spline nonparametric regression model applied to binary response data. A comparative analysis will be conducted between the performance of the Truncated Spline nonparametric regression model for binary response and binary logistic regression model using real data applications as a basis for evaluating the development of the model.

## Method details

To obtain the estimator of multivariable linear Truncated Spline nonparametric regression on data with binary response first by building a linear Truncated Spline nonparametric regression model with optimal knot points. Next, construct the Log Likelihood function and calculate the derivative of each model parameter. Finally, perform numerical iterations using the Newton-Raphson iteration method.

### Probability distribution

Given $x_{1i},x_{2i},\cdot ,x_{pi};i=1,\;2,\cdot ,n$, are as many as p predictor variables. Furthermore, response variable $\left(Y_{i}\right)$ is bernoulli distributed, with a probability distribution of [24]:

$$Y_{i}\;∼\;B\left(1,\pi \left(x_{1i},\;x_{2i},\;\cdot \;,\;x_{pi}\right)\right),\;i=1,2,\cdot ,n$$

with the probability function [24]:

$$P\left(Y_{i}\;=\;y_{i}\right)=\;\pi {\left(x_{i}\right)}^{y_{i}}{\left(1-\pi \left(x_{i}\right)\right)}^{1-y_{i}};\;y_{i}=0,1;\;i=1,2,\cdot ,n$$

Where $\pi (x_{i})$ is defined in the probability distribution function $P(Y_{i}\;=\;y_{i})$ as follows:

(1) $$\begin{array}{ccc} P\left(Y_{i}=\;y_{i}\right) & = & \pi {\left(x_{i}\right)}^{y_{i}}{\left(1-\pi \left(x_{i}\right)\right)}^{1-y_{i}}=\pi {\left(x_{i}\right)}^{y_{i}}{\left(1-\pi \left(x_{i}\right)\right)}^{1}{\left(1-\pi \left(x_{i}\right)\right)}^{-y_{i}}\\ & = & {\left(\frac{\pi \left(x_{i}\right)}{1-\pi \left(x_{i}\right)}\right)}^{y_{i}}\left(1-\pi \left(x_{i}\right)\right) \end{array}$$

### Logit function

In the context of regression for binary response data, the logit function is a tool to transform the nonlinear relationship between predictor variables and probabilities into a linear relationship. This is necessary because the model operates on binary data where the observed response is 0 or 1, and $\pi (x_{i})$ is expressed in non-linear form. Thus, we avoid the problem of limitations in the direct interpretation of $\pi (x_{i})$, since the value of $\pi (x_{i})$ is limited between 0 and 1. Once logarithmized, the logit function can take values over an infinite range.

From Eq. (1), then we made in the natural logarithm function (ln)

(2) $$\ln P\left(Y_{i}\;=\;y_{i}\right)=\;y_{i}\;\ln{\left(\frac{\pi \left(x_{i}\right)}{1-\pi \left(x_{i}\right)}\right)}^{\;}+\ln\left(1-\pi \left(x_{i}\right)\right)$$

When made in exponential form, Eq. (2) forms an exponential family distribution function

$$\exp\left(\ln P\left(Y_{i}\;=\;y_{i}\right)\right)\;=\exp\left(y_{i}\;\ln{\left(\frac{\pi \left(x_{i}\right)}{1-\pi \left(x_{i}\right)}\right)}^{\;}+\ln\left(1-\pi \left(x_{i}\right)\right)\right)$$

where, the distribution function of the exponential family is defined as follows:

$$f\left(y_{i},\;z\right)=\exp\left(\frac{y_{i}.z-b{\left(z\right)}^{\;}}{a\left(\emptyset \right)}+c\left(z,\emptyset \right)\right)$$

Thus,

$$P\left(Y_{i}\;=\;y_{i}\right)=\exp\left(\frac{y_{i}\;\ln{\left(\frac{\pi \left(x_{i}\right)}{1-\pi \left(x_{i}\right)}\right)}^{\;}-\left(-\ln\left(1-\pi \left(x_{i}\right)\right)\;\right)}{1}\right)$$

where, the logit function is obtained

(3) $$z=\ln\left(\frac{\pi \left(x_{i}\right)}{1-\pi \left(x_{i}\right)}\right)$$

Eq. (3) is a link function used to simplify the logistic regression model to facilitate parameter estimation. To achieve this goal, logit transformation is used.

*Logit Transformation Model*

$$z=\ln\left(\frac{\pi \left(x_{i}\right)}{1-\pi \left(x_{i}\right)}\right)$$

$$\ln\left(\exp\left(z\right)\right)=\ln\left(\frac{\pi \left(x_{i}\right)}{1-\pi \left(x_{i}\right)}\right)$$

$$\exp\left(z\right)=\frac{\pi \left(x_{i}\right)}{1-\pi \left(x_{i}\right)}$$

$$\exp\left(z\right)=\pi \left(x_{i}\right)+\exp\left(z\right)\pi \left(x_{i}\right)$$

(4) $$\pi \left(x_{i}\right)=\frac{\exp\left(z\right)}{1+\exp\left(z\right)}$$

The Logistic Regression model can be written as follows Eq. (4) and logit transformation of $\pi (x_{i})$ is defined as follows:

$$\ln\left(\frac{\pi \left(x_{i}\right)}{1-\pi \left(x_{i}\right)}\right)=f\left(x_{1i},\;\cdot \;,x_{pi}\right)$$

$f(x_{1i},\cdot ,x_{pi})$ is approximated by a Linear Truncated Spline function with knot points K_1j_, K_2j, …,_ *K_rj,_* where *j* is 1, 2, …, p, so that the logit equation is obtained as follows:

$$\ln\left(\frac{\pi \left(x_{i}\right)}{1-\pi \left(x_{i}\right)}\right)=\theta_{0}+\sum_{j=1}^{p}\theta_{j1}x_{ji}+\sum_{j=1}^{p}\sum_{u=1}^{r}\theta_{j\left(1+u\right)}{\left(x_{ji}-K_{ju}\right)}_{+};\;i=1,2,\cdot n$$

where, $\theta_{0},\;\theta_{j1},$ and $\theta_{j(1+u)}$, j=1,2,·,p, u=1,2,·,r are the model parameters in the Linear Truncated Spline function. Truncated function for Spline is defined as follows:

$${\left(x_{ji}-\;K_{ju}\right)}_{+}=\;\left\{ \begin{array}{c} \left(x_{ji}-\;K_{ju}\right),\;x_{ji}\geq \;K_{ju}\;\\ 0,\;\;\;x_{ji}<\;K_{ju} \end{array}\right.$$

The logit function of linear Truncated Spline can be presented in matrix form:

$$\left[\begin{array}{cccccccc} 1 & x_{11} & {\left(x_{11}-K_{11}\right)}_{+} & \cdots & x_{p1} & {\left(x_{p1}-K_{11}\right)}_{+} & \cdots & {\left(x_{p1}-K_{1r}\right)}_{+}\\ 1 & x_{12} & {\left(x_{12}-K_{11}\right)}_{+} & \cdots & x_{p2} & {\left(x_{p2}-K_{11}\right)}_{+} & \cdots & {\left(x_{p2}-K_{1r}\right)}_{+}\\ \vdots & \vdots & \vdots & \ddots & \vdots & \vdots & \ddots & \vdots \\ 1 & x_{1n} & {\left(x_{1n}-K_{11}\right)}_{+} & \cdots & x_{pn} & {\left(x_{pn}-K_{11}\right)}_{+} & \cdots & {\left(x_{pn}-K_{1r}\right)}_{+} \end{array}\right]\;\left[\begin{array}{c} \theta_{0}\\ \theta_{11}\\ \theta_{12}\\ \vdots \\ \theta_{p(1+r)} \end{array}\right]$$

Thus, the multivariable linear Truncated Spline nonparametric regression model for binary response data is obtained as follows:

(5) $$\pi \left(x_{i}\right)=\;\frac{\exp\left(\theta_{0}+\;\sum_{j=1}^{p}\theta_{j1}x_{ji}+\;\sum_{j=1}^{p}\sum_{u=1}^{r}\theta_{j\left(1+u\right)}{\left(x_{ji}-\;K_{ju}\right)}_{+}\right)}{1+\left(\theta_{0}+\;\sum_{j=1}^{p}\theta_{j1}x_{ji}+\;\sum_{j=1}^{p}\sum_{u=1}^{r}\theta_{j\left(1+u\right)}{\left(x_{ji}-\;K_{ju}\right)}_{+}\right)};\;i=1,2,\cdot ,n$$

### Likelihood function

Parameter estimation obtained is θ, where

$$\theta ={\left(\begin{array}{ccccccccccc} \theta_{0} & \theta_{11} & \theta_{12} & \cdot & \theta_{1,\left(1+r\right)} & \vdots & \cdots & \vdots & \theta_{p1} & \cdots & \theta_{p,\left(1+r\right)} \end{array}\right)}^{T}$$

The form using *Maximum Likelihood Estimation* (*MLE*) method as follows:

$$l\left(\theta \right)=\prod_{i=1}^{n}P\left(Y_{i}=y_{i}\right)\;=\prod_{i=1}^{n}\pi {\left(x_{i}\right)}^{y_{i}}{\left(1-\pi \left(x_{i}\right)\right)}^{1-y_{i}}$$

(6) $$\;=\pi {\left(x_{i}\right)}^{\sum_{i=1}^{n}y_{i}}{\left(1-\pi \left(x_{i}\right)\right)}^{n-\sum_{i=1}^{n}y_{i}}$$

Parameter estimation can be done with the Maximum Likelihood Estimation method by maximizing the first derivative of the log likelihood function. The likelihood function is easier to maximize in the form of lnl(θ).

*ln-likelihood Function (* L(θ) *)*

(7) $$\begin{array}{ccc} \text{ln}\;\left[l\left(\theta \right)\right] & = & L\left(\theta \right)=\text{ln}\pi {\left(x_{i}\right)}^{\sum_{i=1}^{n}y_{i}}{\left(1-\pi \left(x_{i}\right)\right)}^{n-\sum_{i=1}^{n}y_{i}}\\ & = & \sum_{i=1}^{n}\left\{y_{i}\ln{\left(\frac{\pi {\left(x_{i}\right)}^{\;}}{1-\pi {\left(x_{i}\right)}^{\;}}\right)}^{\;}+\ln\left[1-\pi {\left(x_{i}\right)}^{\;}\right]\right\}\\ & = & \sum_{i=1}^{n}\left\{y_{i}\left(f\left(x_{1i},\cdot ,x_{pi}\right)\right)-\ln\left[1+\exp(f\left(x_{1i},\cdot ,x_{pi}\right))\right]\right\} \end{array}$$

The estimators $\hat{\theta }$ is obtained by deriving the Eq. (7) partially

Derivation of L(θ) with respect to $\theta_{0}$

$$\frac{\partial L\left(\theta \right)}{\partial \theta_{0}}=\sum_{i=1}^{n}\left\{y_{i}\frac{\partial }{\partial \theta_{0}}f\left(x_{1},..,x_{p}\right)-\;\frac{\partial }{\partial \theta_{0}}\ln\left[1+\text{exp}f\left(x_{1},..,x_{p}\right)\right]\right\}\;$$

$$\;=\sum_{i=1}^{n}\left\{y_{i}\left(1\right)-\;\frac{\exp f\left(x_{1},..,x_{p}\right)}{1+\exp f\left(x_{1},..,x_{p}\right)}\left(1\right)\right\}\;=\;\sum_{i=1}^{n}\left\{y_{i}-\;\pi \left(x_{i}\right)\right\}\;$$

Derivation of L(θ) with respect to $\theta_{11}$

$$\frac{\partial L\left(\theta \right)}{\partial \theta_{11}}=\sum_{i=1}^{n}\left\{y_{i}\frac{\partial }{\partial \theta_{11}}f\left(x_{1},..,x_{p}\right)-\;\frac{\partial }{\partial \theta_{11}}\ln\left[1+\text{exp}f\left(x_{1},..,x_{p}\right)\right]\right\}\;$$

$$\;=\sum_{i=1}^{n}\left\{y_{i}x_{1i}-\;\frac{\exp f\left(x_{1},..,x_{p}\right)}{1+\exp f\left(x_{1},..,x_{p}\right)}x_{1i}\right\}=\sum_{i=1}^{n}\left\{y_{i}x_{1i}-\;\pi \left(x_{i}\right)x_{1i}\right\}\;$$

⋮

Derivation of L(θ) with respect to $\theta_{1,(1+r)}$

$$\frac{\partial L\left(\theta \right)}{\partial \theta_{1,\left(1+r\right)}}=\sum_{i=1}^{n}\left\{y_{i}\frac{\partial }{\partial \theta_{1,\left(1+r\right)}}f\left(x_{1},..,x_{p}\right)-\;\frac{\partial }{\partial \theta_{1,\left(1+r\right)}}\ln\left[1+\text{exp}f\left(x_{1},..,x_{p}\right)\right]\right\}\;$$

$$\;=\sum_{i=1}^{n}\left\{y_{i}{\left(x_{1i}-\;K_{1r}\right)}_{+}-\;\frac{\exp f\left(x_{1},..,x_{p}\right)}{1+\exp f\left(x_{1},..,x_{p}\right)}{\left(x_{1i}-\;K_{1r}\right)}_{+}\right\}\;$$

$$\;=\sum_{i=1}^{n}\left\{y_{i}{\left(x_{1i}-\;K_{1r}\right)}_{+}-\;\pi \left(x_{i}\right){\left(x_{1i}-\;K_{1r}\right)}_{+}\right\}\;$$

⋮

Derivation of L(θ) with respect to $\theta_{p,(1+r)}$

$$\frac{\partial L\left(\theta \right)}{\partial \theta_{p,\left(1+r\right)}}=\sum_{i=1}^{n}\left\{y_{i}\frac{\partial }{\partial \theta_{p,\left(1+r\right)}}f\left(x_{1},..,x_{p}\right)-\;\frac{\partial }{\partial \theta_{p,\left(1+r\right)}}\ln\left[1+\text{exp}f\left(x_{1},..,x_{p}\right)\right]\right\}\;$$

$$=\sum_{i=1}^{n}\left\{y_{i}{\left(x_{pi}-\;K_{pr}\right)}_{+}-\;\frac{\exp f\left(x_{1},..,x_{p}\right)}{1+\exp f\left(x_{1},..,x_{p}\right)}{\left(x_{pi}-\;K_{pr}\right)}_{+}\right\}\;$$

$$=\sum_{i=1}^{n}\left\{y_{i}{\left(x_{pi}-\;K_{pr}\right)}_{+}-\;\pi \left(x_{i}\right){\left(x_{pi}-\;K_{pr}\right)}_{+}\right\}\;$$

The same rule is applied for the derivative of L(θ) with respect to each parameter, so that for the first derivative of L(θ) the general equation is given as follows:

$$\frac{\partial L\left(\theta \right)}{\partial \theta_{0}}=\;\sum_{i=1}^{n}\left\{y_{i}-\;\pi \left(x_{i}\right)\right\}\;$$

$$\frac{\partial L\left(\theta \right)}{\partial \theta_{j1}}=\;\sum_{i=1}^{n}\left\{y_{i}x_{ji}-\;\pi \left(x_{i}\right)x_{ji}\right\}\;$$

$$\frac{\partial L\left(\theta \right)}{\partial \theta_{j,\left(1+u\right)}}=\sum_{i=1}^{n}\left\{y_{i}{\left(x_{ji}-\;K_{ju}\right)}_{+}-\;\pi \left(x_{i}\right){\left(x_{ji}-\;K_{ju}\right)}_{+}\right\}\;$$

The estimator $\hat{\theta }$ will be obtained when the derivative equation is equated to 0. However, the equation obtained is implicit (not close form), so it is necessary to continue with numerical iteration. Numerical iteration is performed using the Newton Raphson method with the following given equation [25]:

(8) $$\theta_{\left(t+1\right)}=\theta_{\left(t\right)}-{\left(H_{\left(t\right)}\right)}^{-1}g_{\left(t\right)}$$

Where $\theta_{\left(t+1\right)}$ and $\theta_{\left(t\right)}$ are t-th iteration of parameter value, *t* *=* *1,2,…,*converged. $g_{\left(t\right)}$ is vector with first derivative of ln likelihood function and $H_{\left(t\right)}$ is hessian matrix with second derivative of ln likelihood function, which is given in the following equations:

$$g_{\left(t\right)}=\;\left(\frac{\partial L\left(\theta \right)}{\partial \theta_{0}},\frac{\partial L\left(\theta \right)}{\partial \theta_{11}},\cdots ,\frac{\partial L\left(\theta \right)}{\partial \theta_{1,\left(1+r\right)}},\cdots ,\frac{\partial L\left(\theta \right)}{\partial \theta_{p1}},\frac{\partial L\left(\theta \right)}{\partial \theta_{p2}},\cdots ,\frac{\partial L\left(\theta \right)}{\partial \theta_{p,\left(1+r\right)}}\right)$$

$$H_{\left(t\right)}\;=\left[\begin{array}{cc} \begin{array}{cc} \frac{\partial^{2}L\left(\theta \right)}{\partial {\theta_{0}}^{2}} & \frac{\partial^{2}L\left(\theta \right)}{\partial \theta_{0}\partial \theta_{11}}\\ \frac{\partial^{2}L\left(\theta \right)}{\partial \theta_{11}\partial \theta_{0}} & \frac{\partial^{2}L\left(\theta \right)}{\partial {\theta_{11}}^{2}} \end{array} & \begin{array}{cc} \cdots & \frac{\partial^{2}L\left(\theta \right)}{\partial \theta_{0}\partial \theta_{p,\left(1+r\right)}}\\ \cdots & \frac{\partial^{2}L\left(\theta \right)}{\partial \theta_{11}\partial \theta_{p,\left(1+r\right)}} \end{array}\\ \begin{array}{cc} \vdots & \vdots \\ \frac{\partial^{2}L\left(\theta \right)}{\partial \theta_{p,\left(1+r\right)}\partial \theta_{0}} & \frac{\partial^{2}L\left(\theta \right)}{\partial \theta_{p,\left(1+r\right)}\partial \theta_{11}} \end{array} & \begin{array}{cc} \ddots & \vdots \\ \cdots & \frac{\partial^{2}L\left(\theta \right)}{\partial {\theta_{p,\left(1+r\right)}}^{2}} \end{array} \end{array}\right]$$

$\hat{\theta }$ will obtained when [25]

$$\left|\theta_{\left(t+1\right)}-\;\theta_{\left(t\right)}\right|<\;\varepsilon ,\varepsilon =0.000001$$

Based on the Eq. (7) will be performed the second derivative of L(θ)

Derivation of L(θ) with respect to $\theta_{0}$ and $\theta_{0}$

$$\frac{\partial L\left(\theta \right)}{\partial {\theta_{o}}^{2}}=\sum_{i=1}^{n}\left\{\frac{\partial }{\partial \theta_{0}}y_{i}-\;\frac{\partial }{\partial \theta_{0}}\frac{\exp f\left(x_{1},..,x_{p}\right)}{1+\exp f\left(x_{1},..,x_{p}\right)}\right\}\;$$

$$=\sum_{i=1}^{n}\left\{0-\frac{\left[\exp f\left(x_{1},..,x_{p}\right)\right]\left[1+\exp f\left(x_{1},..,x_{p}\right)\right]-\left[\exp f\left(x_{1},..,x_{p}\right)\right].\;\left[\exp f\left(x_{1},..,x_{p}\right)\right]}{{\left[1+\exp f\left(x_{1},..,x_{p}\right)\right]}^{2}}\right\}\;$$

$$=\sum_{i=1}^{n}\left\{0-\;\frac{\left[\text{exp}f\left(x_{1},..,x_{p}\right)\right]}{\left[1+\text{exp}f\left(x_{1},..,x_{p}\right)\right]}\;\frac{1}{\left[1+\text{exp}f\left(x_{1},..,x_{p}\right)\right]}\right\}=\;-\;\sum_{i\;=\;1}^{n}\;\pi \left(x_{i}\right)\;\left(1-\pi \left(x_{i}\right)\right)\;$$

⋮

Derivation of L(θ) with respect to $\theta_{11}$ and $\theta_{12}$

$$\frac{\partial L\left(\theta \right)}{\partial \theta_{12}\partial \theta_{11}}=\sum_{i=1}^{n}\left\{\frac{\partial }{\partial \theta_{12}}y_{i}x_{1i}-\;\frac{\partial }{\partial \theta_{12}}\frac{\exp f\left(x_{1},..,x_{p}\right)}{1+\exp f\left(x_{1},..,x_{p}\right)}x_{1i}\right\}\;$$

$$=\sum_{i=1}^{n}\left\{0-\;\frac{\left[\exp f\left(x_{1},..,x_{p}\right)\right]\left[1+\exp f\left(x_{1},..,x_{p}\right)\right]-\left[\exp f\left(x_{1},..,x_{p}\right)\right].\;\left[\exp f\left(x_{1},..,x_{p}\right)\;\right]}{{\left[1+\exp f\left(x_{1},..,x_{p}\right)\right]}^{2}}{\left(x_{1i}-\;K_{11}\right)}_{+}x_{1i}\right\}\;$$

$$=\sum_{i=1}^{n}\left\{0-\;\frac{\left[\text{exp}f\left(x_{1},..,x_{p}\right)\right]}{\left[1+\text{exp}f\left(x_{1},..,x_{p}\right)\right]}\;\frac{1}{\left[1+\text{exp}f\left(x_{1},..,x_{p}\right)\right]}{\left(x_{1i}-\;K_{11}\right)}_{+}x_{1i}\right\}\;$$

$$=-\;\sum_{i\;=\;1}^{n}\;\pi \left(x_{i}\right)\;\left(1-\pi \left(x_{i}\right)\right){\left(x_{1i}-\;K_{11}\right)}_{+}x_{1i}$$

⋮

Derivation of L(θ) with respect to $\theta_{p,(1+r)}$ and $\theta_{q,(1+r)}$

$$\frac{\partial L\left(\theta \right)}{\partial \theta_{q,\left(1+r\right)}\partial \theta_{p,\left(1+r\right)}}=\sum_{i=1}^{n}\left\{\frac{\partial }{\partial \theta_{q,\left(1+r\right)}}y_{i}{\left(x_{pi}-\;K_{pr}\right)}_{+}-\;\frac{\partial }{\partial \theta_{q,\left(1+r\right)}}\frac{\exp f\left(x_{1},..,x_{p}\right)}{1+\exp f\left(x_{1},..,x_{p}\right)}{\left(x_{pi}-\;K_{pr}\right)}_{+}\right\}\;$$

$$=\sum_{i=1}^{n}\left\{0-\;\frac{\left[\exp f\left(x_{1},..,x_{p}\right)\right]\left[1+\exp f\left(x_{1},..,x_{p}\right)\right]-\left[\exp f\left(x_{1},..,x_{p}\right)\right].\;\left[\exp f\left(x_{1},..,x_{p}\right)\;\right]}{{\left[1+\exp f\left(x_{1},..,x_{p}\right)\right]}^{2}}{\left(x_{qi}-\;K_{qr}\right)}_{+}{\left(x_{pi}-\;K_{pr}\right)}_{+}\right\}\;$$

$$=\sum_{i=1}^{n}\left\{0-\;\frac{\left[\exp f\left(x_{1},..,x_{p}\right)\right]}{\left[1+\exp f\left(x_{1},..,x_{p}\right)\right]}\;\frac{1}{\left[1+\exp f\left(x_{1},..,x_{p}\right)\right]}{\left(x_{qi}-\;K_{qr}\right)}_{+}{\left(x_{pi}-\;K_{pr}\right)}_{+}\right\}\;$$

$$=-\;\sum_{i\;=\;1}^{n}\;\pi \left(x_{i}\right)\;\left(1-\pi \left(x_{i}\right)\right){\left(x_{qi}-\;K_{qr}\right)}_{+}{\left(x_{pi}-\;K_{pr}\right)}_{+}$$

Thus, the estimator $\hat{\theta }$ obtained is

$$\hat{\theta }={\left(\begin{array}{cccccccccc} {\hat{\theta }}_{0} & {\hat{\theta }}_{11} & \cdot & {\hat{\theta }}_{1,\left(1+r\right)} & \vdots & \cdots & \vdots & {\hat{\theta }}_{p1} & \cdots & {\hat{\theta }}_{p,\left(1+r\right)} \end{array}\right)}^{T}$$

So the model can be written as follows:

$$\hat{\pi }\left(x_{i}\right)=\;\frac{\exp\left({\hat{\theta }}_{0}+\;\sum_{j=1}^{p}{\hat{\theta }}_{j1}x_{ji}+\;\sum_{j=1}^{p}\sum_{u=1}^{r}{\hat{\theta }}_{j\left(1+u\right)}{\left(x_{ji}-\;K_{ju}\right)}_{+}\right)}{1+\left({\hat{\theta }}_{0}+\;\sum_{j=1}^{p}{\hat{\theta }}_{j1}x_{ji}+\;\sum_{j=1}^{p}\sum_{u=1}^{r}{\hat{\theta }}_{j\left(1+u\right)}{\left(x_{ji}-\;K_{ju}\right)}_{+}\right)};\;i=1,2,\cdot ,n$$

with $K_{ju}$ is knot points.

### Optimal knot points

The optimal knot points for the linear Truncated Spline nonparametric regression model can be determined based on the minimum Akaike Information Criterion (AIC) value. To calculate the AIC value is given in the following equation [26]:

(9) AIC=2k−2L(θ)

Where k is number of parameters in the model and L(θ) is likelihood function of the model. The likelihood function in generally given in the following Eq. (7). In the context of truncated spline regression, knot points are used to determine where the spline function changes locally. Each predictor variable can have different knot points. So the AIC equation can be written as follows

(10) $$\text{AIC}\left(K_{jr}\right)=\left(2\left(\sum_{j=1}^{p}u_{j}+p\;+\;1\right)\right)-2L\left(\theta \right)$$

Where j=1,2,·,p, and $u_{j}$ is number of knot points on the j-th predictor.

Suppose given predictor variables $x_{1i},\;x_{2i},$ and $x_{3i}$. If $u_{1}$= 1, $u_{2}$ = 1, and $u_{3}$=1. Then the equation for calculating AIC value is as follows

$$\text{AIC}\left(K_{jr}\right)=\;14-2L\left(\theta \right)$$

⋮

Suppose given predictor variables $x_{1i},x_{2i},$ and $x_{3i}$. If $u_{1}$= 1, $u_{2}$ = 3, and $u_{3}$=2. Then the equation for calculating AIC value is as follows

$$\text{AIC}\left(K_{jr}\right)=\;20-2L\left(\theta \right)$$

⋮

Suppose given predictor variables $x_{1i},x_{2i},$ and $x_{3i}$. If $u_{1}$= 3, $u_{2}$ = 3, and $u_{3}$=3. Then the equation for calculating AIC value is as follows

$$\text{AIC}\left(K_{jr}\right)=\;26-2L\left(\theta \right)$$

## Method validation

**Fig. 1:**
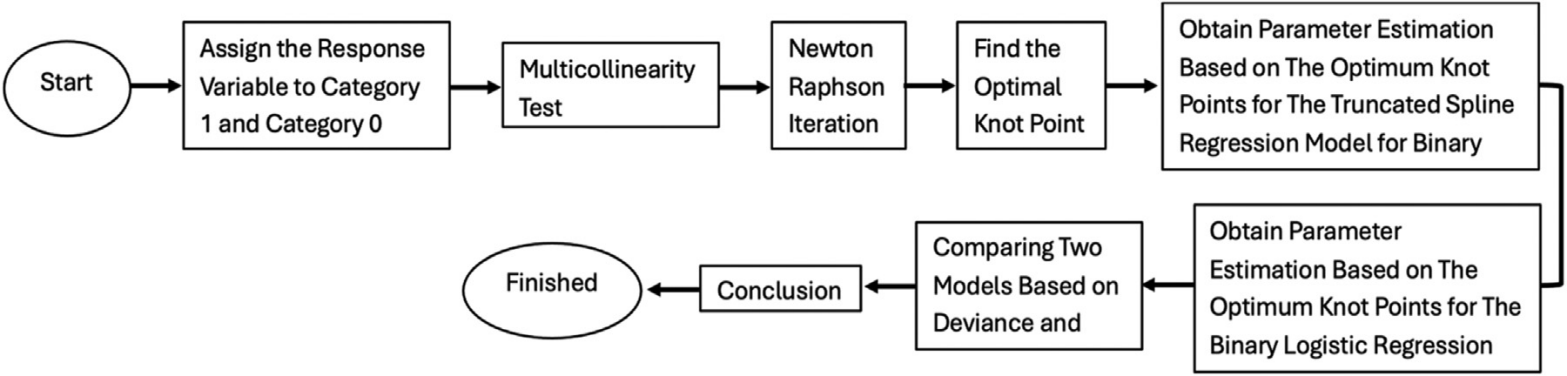
Flowchart of methodology.

In applying the multivariable linear Truncated Spline nonparametric regression method for binary response data, we used 2 application data: achievement status data of unmet need targets in East Java, Indonesia in 2023 and percentage of poor population in Indonesia in 2023. Fig. 1 presents a flowchart illustrating the overall methodology of this study for real data application.

### Real data application

**Case 1: Achievement Status Data of Unmet Need Targets in East Java, Indonesia**

We used achievement status data of unmet need targets in East Java, Indonesia which consists of 1 response variable and 3 predictor variables that are thought to affect the response variable. The whole data is collected from publicatin of BKKBN official website. The detail of the variables are described in Table 1 .

**Table 1:** Variable desciption.

Variabel	Notation	Descipriton
Response	y	1 = unmet need target not achieved 0 = unmet need target achieved
Predictor	$\;X_{1}$	Percentage of family heads with no primary education
	$\;X_{2}$	Percentage of couples of childbearing age (PUS) with 2 children
	$\;X_{3}$	Percentage of couples of childbearing age (PUS) who seek services at FKTP (Health centers or equivalent, doctor's practices, private clinics or equivalent and class D private hospitals or equivalent)

**Fig. 2:**
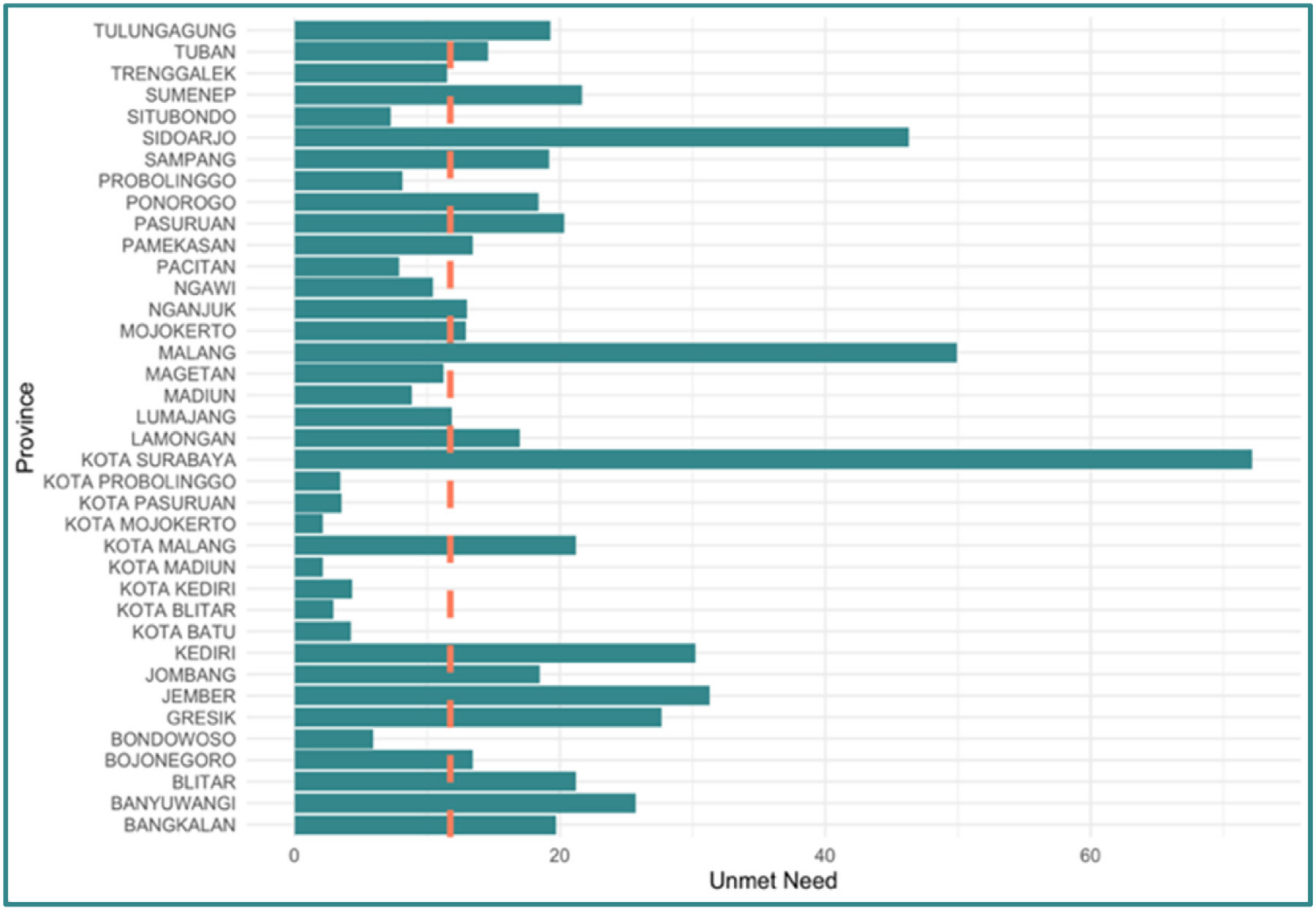
Categories of achievement status data of unmet need target in east java.

Fig. 2 provides information about response variable. The response data is categorized using the target value of unmet need in East Java Province, which is 11.74 %. Based on this data, there are 23 districts/cities where the unmet need target status has not been achieved which will be categorized as 1 and 15 districts/cities where the unmet need target status has been achieved which will be categorized as 0.

### Descriptive analysis

Descriptive analysis is used to characterize the data for each variable shown in the descriptive analysis results as follows:

Table 2 provides information about the characteristics of the variables. In addtion, it is obtained that no multicollinearity between the predictor variables.The characteristics data for each variables shown thorugh in below:

**Table 2:** Desciptive statistics of research variables.

Variable	Category	Mean	Median	Minimum	Maximum
$\;X_{1}$	1	9.562	7.830	1.810	30.30
	0	7.755	4.770	1.610	19.800
$\;X_{2}$	1	41.98	43.46	33.34	46.48
	0	41.53	41.66	37.49	44.54
$\;X_{3}$	1	78.12	78.32	57.31	91.11
	0	70.65	74.49	46.91	87.91

**Fig. 3:**
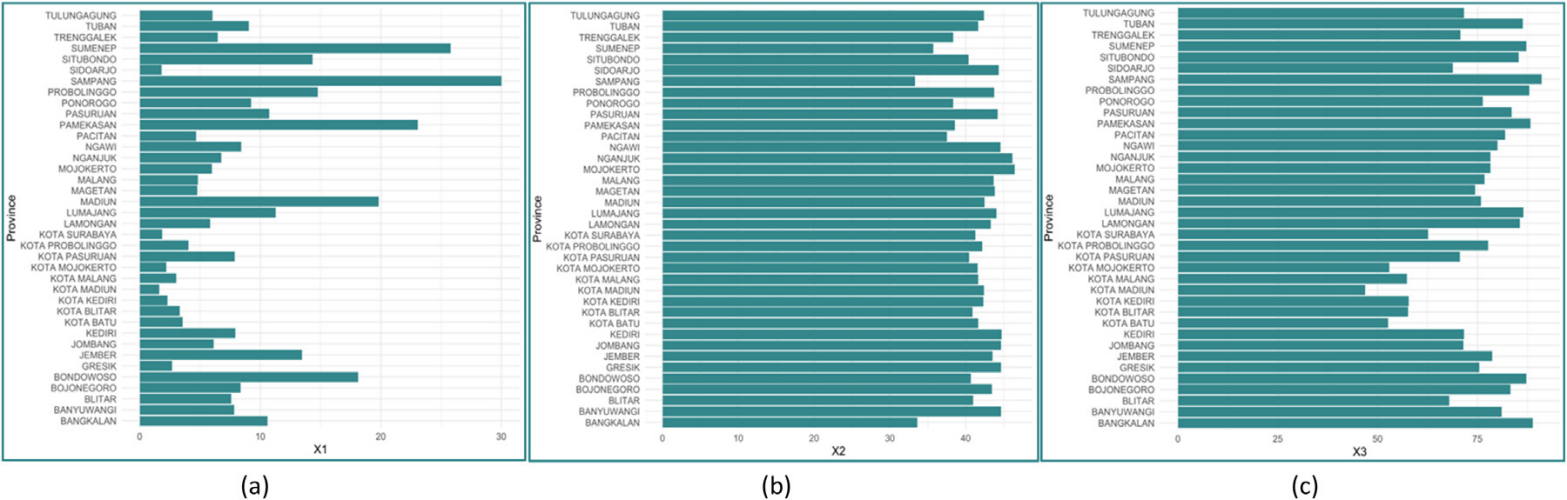
Characteristics of each predictor.

**Fig. 4:**
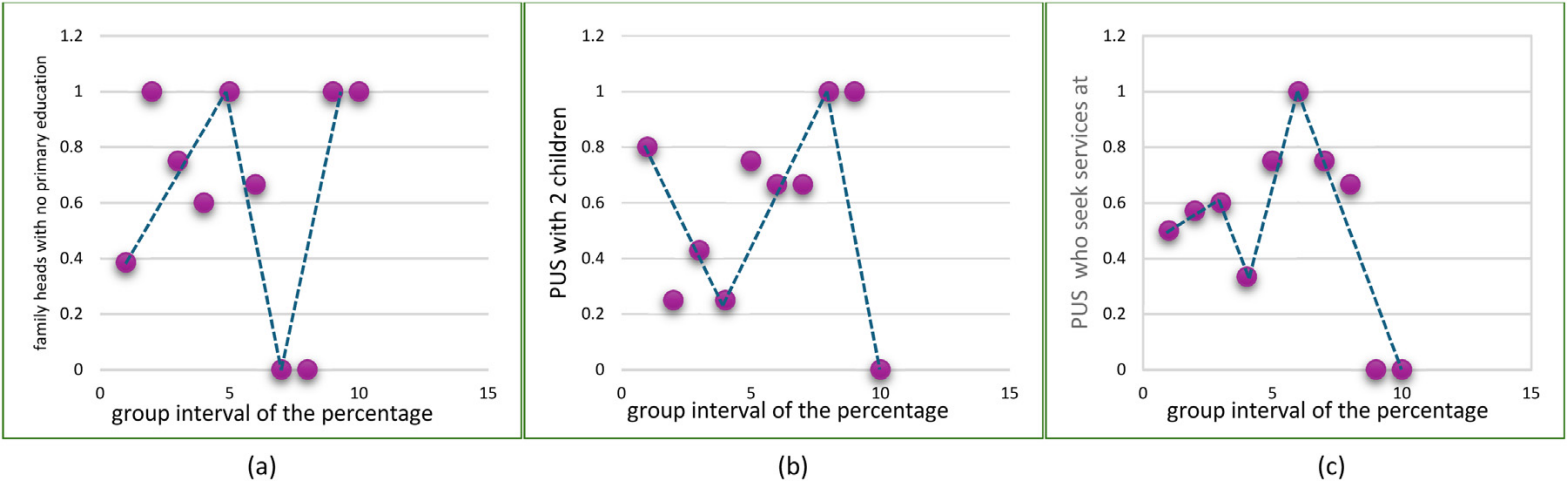
Scatterplots of Variable Each Group.

Based on Fig. 3, (a) it can be seen that Sampang is the highest percentage of family heads with no primary education, and Madiun is the lowest percentage of family heads with no primary education. (b) shows that Mojokerto is the highest percentage of PUS with 2 children, and Sampang is the lowest percentage of PUS with 2 children, and (c) shows that Sampang is the highest percentage of PUS who seek services at FKTP, and Madiun is the lowest percentage of PUS who seek services at FKTP. The scatterplots data for each predictor variable is shown through plots according [24] in Fig. 4 below:

The scatterplot created for each predictor variable that has been built into several group intervals. Based on (a) the percentage of family heads with no primary education in group 5 data has an upward pattern tendency, then between 5 and 8 data patterns tend to decrease, while groups 10 data patterns tend to increase. Based on (b) Percentage of couples of childbearing age (PUS) with 2 children in group 5 data has a downward pattern tendency, then between 5 and 9 data patterns tend to increase, while above 10 data patterns tend to decrease again. Based on (c) Percentage of couples of childbearing age (PUS) who seek services at FKTP in group 4 data has a tendency for the pattern to increase, then in group 5 the data pattern decreases, then the data pattern tends to increase in group 8 data, and group data above 8 data patterns tend to fall back. Based on the pattern in Fig. 4, it can be assumed that the data pattern changes at certain sub-intervals, so this case is suitable for using a linear truncated spline.

### Linear truncated spline nonparametric regression model

Based on Eq. (5), the linear Truncated Spline nonparametric regression model for achievement status data of target unmet need in East Java is as follows:

$$\pi \left(x_{i}\right)=\;\frac{\exp\left(\theta_{0}+\;\theta_{11}x_{1i}+\theta_{21}x_{2i}+\theta_{31}x_{3i}+\;\sum_{j=1}^{3}\sum_{u=1}^{r}\theta_{j\left(1+u\right)}{\left(x_{ji}-\;K_{ju}\right)}_{+}\;\right)}{1+\exp\left(\theta_{0}+\;\theta_{11}x_{1i}+\theta_{21}x_{2i}+\theta_{31}x_{3i}+\;\sum_{j=1}^{3}\sum_{u=1}^{r}\theta_{j\left(1+u\right)}{\left(x_{ji}-\;K_{ju}\right)}_{+}\;\right)\;}$$

### Selecting the optimal knots point

To obtain the optimal model, researchers used a combination of the number of knot points limited to 3. The knot point locations and AIC values obtained from the model are given in Table 3. Based on the table 3, the optimal knot points obtained are 8.715, 12.267, and 19.372 for $X_{1}$, 41.552, and 43.195 for $X_{2}$, then 63.485, and 80.060 for $X_{3}$ is a model with an minimum AIC value of 30.979.

**Table 3:** AIC value based on knot point candidate.

Number of Knot Points	$K_{1u}$			$K_{2u}$			$K_{3u}$			AIC (K)
	$K_{11}$	$K_{12}$	$K_{13}$	$K_{21}$	$K_{22}$	$K_{23}$	$K_{31}$	$K_{32}$	$K_{33}$	
1,1,1	22.925			34.925			63.485			46.397
1,2,2	22.925			39.910	41.552		63.485	74.535		47.723
3,1,3	8.715	12.267	19.372	41.552			69.010	74.535	80.060	31.720
⋮										
2,2,2	12.267	22.925		39.910	41.552		63.485	80.060		41.218
**3,2,2**	**8.715**	**12.267**	**19.372**	**41.552**	**43.195**		**63.485**	**80.060**		30.979*
⋮										
3,3,3	8.715	12.267	19.372	41.552	43.195	44.837	69.010	74.535	80.060	34.219

The linear Truncated Spline nonparametric regression model based on the optimal knot points is given as follows:

$$\pi \left(x_{i}\right)=\frac{\exp\left(z\right)}{1+\exp\left(z\right)}$$

Where

$$\begin{array}{ccc} z & = & \theta_{0}+\theta_{11}x_{1i}+\theta_{12}{\left(x_{1i}-\;8.715\right)}_{+}+\theta_{13}{\left(x_{1i}-\;12.267\right)}_{+}+\theta_{14}{\left(x_{3i}-\;19.372\right)}_{+}+\theta_{21}x_{2i}+\theta_{22}{\left(x_{2i}-\;41.552\right)}_{+}\\ & & +\;\theta_{23}{\left(x_{2i}-\;43.195\right)}_{+}+\theta_{31}x_{3i}+\theta_{32}{\left(x_{3i}-\;63.485\right)}_{+}+\theta_{33}{\left(x_{3i}-\;80.060\right)}_{+} \end{array}$$

### Parameter estimation results of multivariable linear truncated spline nonparametric regression model

With the linear Truncated Spline nonparametric regression method, the model parameter estimation results for achievement status data of target unmet need in East Java are given in the following table:

Based on the estimation results in Table 4, the Truncated Spline nonparametric linear regression model is given as follows:

$$\pi \left(x_{i}\right)=\frac{\exp\left(z\right)}{1+\exp\left(z\right)}$$

**Table 4:** Parameter Estimation Results.

Parameters	Estimations	Parameters	Estimations
$\theta_{0}$	−10.970	$\theta_{22}$	2.146
$\theta_{11}$	−0.022	$\theta_{23}$	−1.032
$\theta_{12}$	1.345	$\theta_{31}$	0.634
$\theta_{13}$	−4.256	$\theta_{33}$	−0.958
$\theta_{14}$	7.090	$\theta_{33}$	0.755
$\theta_{21}$	−0.650		

Where

$$\begin{array}{ccc} z & = & -10.970-0.022x_{1i}+1.345{\left(x_{1i}-\;8.715\right)}_{+}-4.256{\left(x_{1i}-\;12.267\right)}_{+}+7.090{\left(x_{3i}-\;19.372\right)}_{+}-0.650x_{2i}+2.146{\left(x_{2i}-\;41.552\right)}_{+}\\ & & -\;1.032{\left(x_{2i}-\;43.195\right)}_{+}+0.634x_{3i}-0.958{\left(x_{3i}-\;63.485\right)}_{+}+0.755{\left(x_{3i}-\;80.060\right)}_{+} \end{array}$$

### Parameter estimation results of binary logistic regression model

The binary logistic regression model for achievement status data of target unmet need in East Java is as follows:

$$\pi \left(x_{i}\right)=\;\frac{\text{exp}\left(-8.421-0.025x_{1i}-0.081x_{2i}\;+0.076x_{3i}\right)}{1+\text{exp}\left(-8.421-0.025x_{1i}-0.081x_{2i}\;+0.076x_{3i}\right)}$$

**Case 2: Percentage of Poor Population in Indonesia**

We used percentage of poor population data in Indonesia which consists of 1 response variable and 3 predictor variables that are thought to affect the response variable. The whole data is collected from publicatin of BPS official website. The detail of the variables are described in Table 5.

**Table 5:** Variable Desciption.

Variabel	Notation	Descipriton
Response	y	1 = high percentage of poor population 0 = low percentage of poor population
Predictor	$\;X_{1}$	Average Years of Schooling
	$\;X_{2}$	Labor Force Participation Rate
	$\;X_{3}$	Expected Years of Schooling

**Fig. 5:**
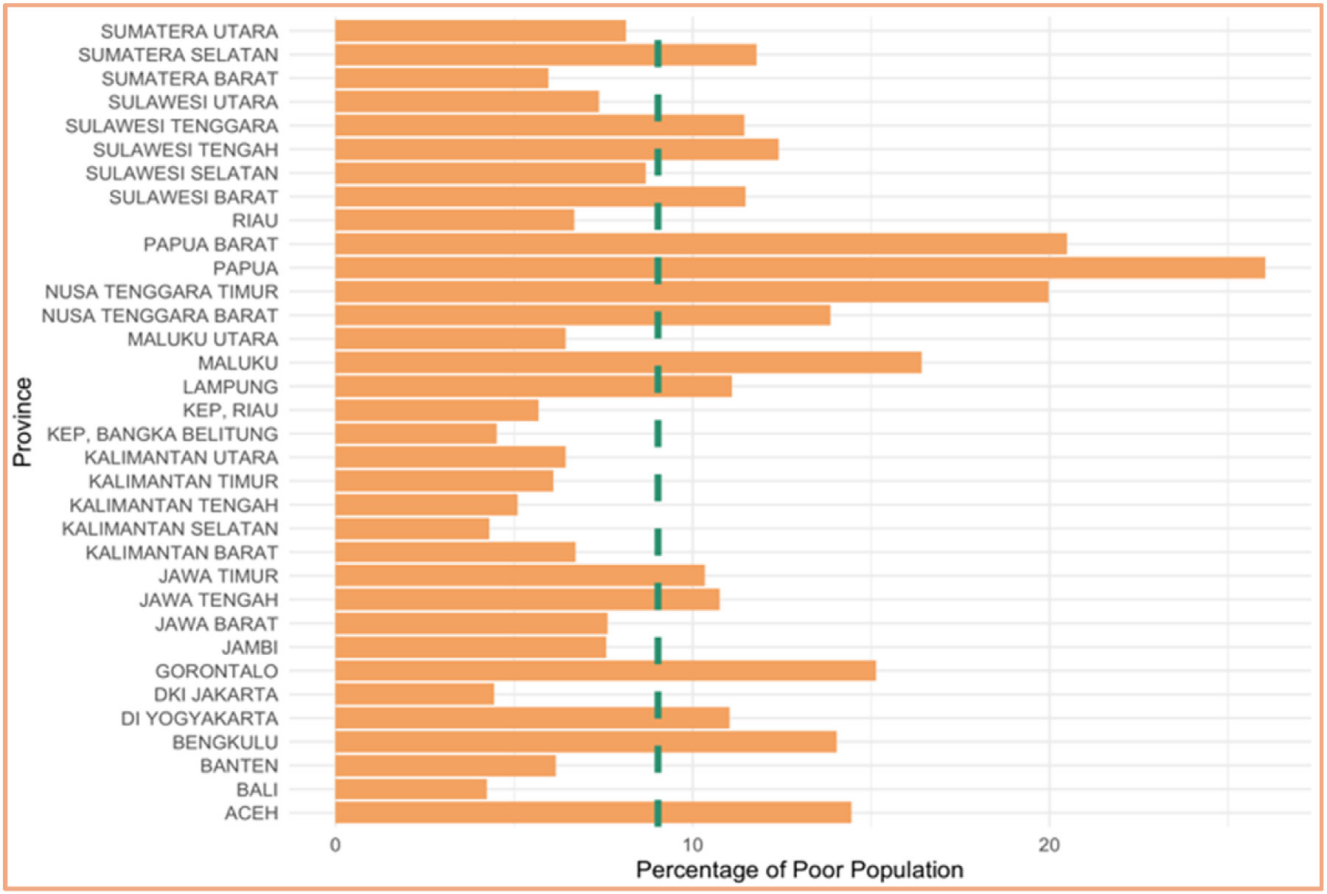
Categories of percentage of poor population.

Fig. 5 provides information about response variable. The response data is categorized using the percentage of poor population in Indonesia, which 9.03. Based on this data, there are 16 provinces that have a high percentage of poor population which will be categorized as 1 and 18 provinces that have a low percentage of poor population which will be categorized as 0.

### Descriptive analysis

Descriptive analysis is used to characterize the data for each variable shown in the descriptive analysis results as follows:

Table 6 provides information about the characteristics of the variables. In addtion, it is obtained that no multicollinearity between the predictor variables. The characteristics data for each variables shown thorugh in below:

**Table 6:** Desciptive statistics of research variables.

Variable	Category	Mean	Median	Minimum	Maximum
$\;X_{1}$	1	8.977	8.795	7.150	11.540
	0	8.785	8.855	7.710	10.200
$\;X_{2}$	1	5.010	4.885	3.040	7.890
	0	4.624	4.290	3.210	7.970
$\;X_{3}$	1	13.39	13.34	11.15	15.66
	0	13.21	13.16	12.31	14.11

**Fig. 6:**
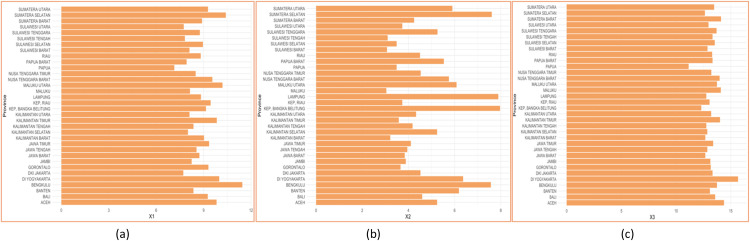
Characteristics of each predictor.

**Fig. 7:**
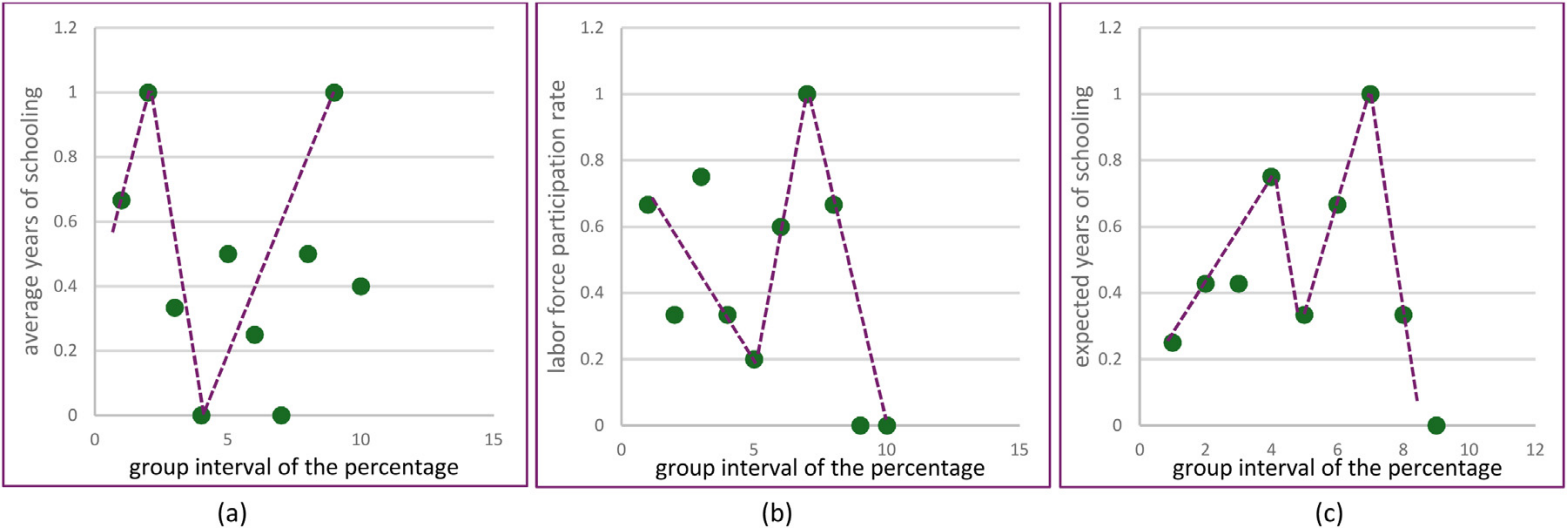
Scatterplots of variable each group.

Based on Fig. 6, (a) it can be seen that Bengkulu is the highest average years of schooling, and Papua is the lowest highest average years of schooling. (b) shows that Kep. Bangka Belitung is the highest labor force participation rate, and Maluku is the lowest labor force participation rate, and (c) shows that DI Yogyakarta is the highest expected years of schooling, and Papua is the lowest expected years of schooling. The scatterplots data for each predictor variable is shown through plots according [24] in Fig. 7 below

The scatterplot created for each predictor variable that has been built into several group intervals. Based on (a) the average years of schooling in group 2 data has an upward pattern tendency, then data group 4 patterns tend to decrease, while above groups 5 data patterns tend to increase. Based on (b) labor force pariticipation rate in group 5 data has a downward pattern tendency, then between 5 and 8 data patterns tend to increase, while above 10 data patterns tend to decrease again. Based on (c) expected years of schooling in group 3 data has a tendency for the pattern to increase, then in group 5 the data pattern decreases, then the data pattern tends to increase in group 8 data, and group data above 8 data patterns tend to fall back. Based on the pattern in Fig. 7, it can be assumed that the data pattern changes at certain sub-intervals, so this case is suitable for using a linear truncated spline.

#### Linear truncated spline nonparametric regression model

Based on Eq. (5), the linear Truncated Spline nonparametric regression model for percentage of poor population in Indonesia is as follows:

$$\pi \left(x_{i}\right)=\;\frac{\exp\left(\theta_{0}+\;\theta_{11}x_{1i}+\theta_{21}x_{2i}+\theta_{31}x_{3i}+\;\sum_{j=1}^{3}\sum_{u=1}^{r}\theta_{j\left(1+u\right)}{\left(x_{ji}-\;K_{ju}\right)}_{+}\;\right)}{1+\exp\left(\theta_{0}+\;\theta_{11}x_{1i}+\theta_{21}x_{2i}+\theta_{31}x_{3i}+\;\sum_{j=1}^{3}\sum_{u=1}^{r}\theta_{j\left(1+u\right)}{\left(x_{ji}-\;K_{ju}\right)}_{+}\;\right)\;}$$

### Selecting the optimal knots point

Based on the table 7, the optimal knot points obtained are 8.583 for $X_{1}$, 4.683, 5.505, and 6.327 for $X_{2}$, then 11.901, and 12.653 for $X_{3}$ is a model with an minimum AIC value of 47.221.

**Table 7:** AIC Value based on Knot Point Candidate.

Number of Knot Points	$K_{1u}$			$K_{2u}$			$K_{3u}$			AIC (K)
	$K_{11}$	$K_{12}$	$K_{13}$	$K_{21}$	$K_{22}$	$K_{23}$	$K_{31}$	$K_{32}$	$K_{33}$	
1,1,1	10.017			3.862			11.901			51.351
1,2,2	7.867			6.327	7.148		11.901	14.156		49.336
3,1,3	7.867	8.583	9.300	6.327			11.901	12.653	14.156	52.748
⋮										
**1,3,2**	**8.583**			**4.683**	**5.505**	**6.327**	**11.901**	**12.653**		47.221*
3,2,3	7.867	9.300	10.017	3.861	6.327		11.901	12.653	14.157	53.537
⋮										
3,3,3	8.583	9.300	10.017	4.683	5.505	6.327	11.901	13.405	14.157	50.966

The linear Truncated Spline nonparametric regression model based on the optimal knot points is given as follows:

$$\pi \left(x_{i}\right)=\frac{\exp\left(z\right)}{1+\exp\left(z\right)}$$

Where

$$\begin{array}{ccc} z & = & \theta_{0}+\theta_{11}x_{1i}+\theta_{12}{\left(x_{1i}-\;8.583\right)}_{+}+\theta_{21}x_{2i}+\theta_{22}{\left(x_{2i}-\;4.683\right)}_{+}+\theta_{23}{\left(x_{2i}-\;5.505\right)}_{+}+\theta_{24}{\left(x_{2i}-\;6.327\right)}_{+}\\ & & +\;\theta_{31}x_{3i}+\theta_{32}{\left(x_{3i}-\;11.901\right)}_{+}+\theta_{33}{\left(x_{3i}-\;12.653\right)}_{+} \end{array}$$

### Parameter Estimation Results of Multivariable Linear Truncated Spline Nonparametric Regression Model

With the linear Truncated Spline nonparametric regression method, the model parameter estimation results for percentage of poor population in Indonesia are given in the following table:

Based on the estimation results in Table 8, the Truncated Spline nonparametric linear regression model is given as follows:

$$\pi \left(x_{i}\right)=\frac{\exp\left(z\right)}{1+\exp\left(z\right)}$$

**Table 8:** Parameter Estimation Results.

Parameters	Estimations	Parameters	Estimations
$\theta_{0}$	−7.249	$\theta_{23}$	−7.944
$\theta_{11}$	−1.005	$\theta_{24}$	7.143
$\theta_{12}$	1.331	$\theta_{31}$	1.760
$\theta_{21}$	−1.274	$\theta_{32}$	−4.404
$\theta_{22}$	4.692	$\theta_{33}$	4.039

Where

$$\begin{array}{ccc} z & = & -7.249-1.005x_{1i}+1.331{\left(x_{1i}-\;8.583\right)}_{+}-1.274x_{2i}+4.692{\left(x_{2i}-\;4.683\right)}_{+}-7.944{\left(x_{2i}-\;5.505\right)}_{+}\\ & & +\;7.143{\left(x_{2i}-\;6.327\right)}_{+}+1.760x_{3i}-4.404{\left(x_{3i}-\;11.901\right)}_{+}+4.039{\left(x_{3i}-\;12.653\right)}_{+} \end{array}$$

### Parameter Estimation Results of Binary Logistic Regression Model

The binary logistic regression model for percentage of poor population in Indonesia is as follows:

$$\pi \left(x_{i}\right)=\;\frac{\text{exp}\left(-5.234-0.031x_{1i}+0.188x_{2i}+0.337x_{3i}\right)}{1+\text{exp}\left(-5.234-0.031x_{1i}+0.188x_{2i}+0.337x_{3i}\right)}$$

### Comparing of Truncated Spline Nonparametric Regression for Binary Response and Binary Logistic Regression

#### Obtaining the Best Model Based on Deviance Value

The regression model chosen is the model with the smallest deviance value. Based on the deviance statistical test, the results obtained are as follows:

Based on Table 9, the deviance value for truncated spline nonparametric regression model for case 1 (29.430) and case 2 (38.327) is smaller than the deviance value for the logistic regression model (46.123 and 45.962). So that the truncated spline nonparametric regression for binary response model is a better model for achievemet status data of unmet need target in East Java, Indonesia and percentage of poor population in Indonesia.

**Table 9:** Comparison of Deviance Values.

Model	Deviance Values	
	Case 1	Case 2
Truncated Spline Nonparametric Regression for Binary Response	29.430	38.327
Binary Logistic Regression	46.123	45.962

#### Obtaining the Best Model Based on Evaluation Criteria Value

Table 10 shows that for case 1 there are 10 districts/cities classified as unmet need target achieved and 21 districts/cities classified as unmet need target not achieved that are correctly predicted by the Truncated Spline Nonparametric regression for binary response model. For the binary logistic regression model, the table shows that there were 6 districts classified as unmet need target achieved, and 20 districts classified as unmet need target not achieved that were correctly predicted by the model. For case 2 there are 15 provincies classified as low percentage of poor population and 11 provinces classified as high percentage of poor population that are correctly predicted by the Truncated Spline Nonparametric regression for binary response model, and the binary logistic regression model shows that there were 13 provinces classified as low percentage of poor population, and 8 provinces classified as high percentage of poor population that are correctly predicted by the model.

**Table 10:** Confusion Matrix.

	Case 1			Case 2		
Truncated Spline Nonparametric Regression for Binary Response	**Prediction**					

	**0**	**1**		**0**	**1**
**Actual**	**0**	10	5	**0**	15	3
	**1**	2	21	**1**	5	11

Binary Logistic Regression	**Prediction**					

	**0**	**1**		**0**	**1**
**Actual**	**0**	6	9	**0**	13	5
	**1**	3	20	**1**	8	8

In evaluating the performance of regression models for binary response data, evaluation criteria are used to determine how well the model can classify the data. Table 11 below presents the comparison between the two regression models.

**Table 11:** Comparison of evaluation criteria value.

**Criteria**	**Case 1**		**Case 2**	
	**Model**			
	**Truncated Spline Nonparametric Regression for Binary Response**	**Binary Logistic Regression**	**Truncated Spline Nonparametric Regression for Binary Response**	**Binary Logistic Regression**
Accuracy	81.58 %	68.42 %	76.47 %	61.76 %
Sensitivity	91.30 %	86.96 %	68.75 %	50 %
Specificity	66.76 %	40 %	83.33 %	72.22 %
Precision	80.77 %	68.97 %	78.57 %	61.54 %

The accuracy value shows the proportion of all correct predictions out of the total observations. From table 11, it can be seen that the Truncated Spline Nonparametric Regression model has a higher accuracy for case 1 and case 2 (81.58 and 76.47 %) than Logistic Regression (68.42 % and 61.76 %). This indicates that this nonparametric model is more reliable in making correct predictions overall. Sensitivity, measures the ability of the model to correctly identify positive cases. The Truncated Spline Nonparametric Regression model showed higher sensitivity for case 1 and case 2 (91.30 % and 68.75 %) than Logistic Regression (86.96 % and 50 %). This means that the nonparametric model is more effective in detecting positive cases. Specificity measures the ability of the model to correctly identify negative cases. The Truncated Spline Nonparametric Regression model also excelled in this criterion for case 1 and case 2, with a specificity value of 66.76 % and 83.33 % compared to only 40 % and 72.22 % in the Logistic Regression model. This suggests that the nonparametric model is better at avoiding mis-detection of cases that are actually negative. The precision value measures the proportion of positive predictions that are actually positive. In this criterion, the Truncated Spline Nonparametric Regression has a precision of 80.77 % for case 1 and 78.57 % for case 2, higher than the Logistic Regression which has a precision of 68.97 % for case 1 and 61.54 % for case 2. This means that the nonparametric model is better at ensuring that positive predictions are more likely to be correct.

Based on the evaluation criteria, overall the Truncated Spline Nonparametric Regression model for binary response data performed better than the Binary Logistic Regression. This nonparametric model showed advantages in accuracy, sensitivity, specificity and precision, making it a more appropriate choice for the analysis of data with similar pattern data characteristics.

## Conclusion

Truncated spline nonparametric regression was developed to model the relationship between binary response variables and predictor variables that have changing patterns at certain sub-intervals. This model uses the Bernoulli distribution approach and is built based on the Maximum Likelihood Estimation (MLE) method. The main advantage of this model is its flexibility in capturing nonlinear relationships between variables, resulting in a more accurate estimation without imposing a particular functional form. The generalization of this model can be applied to other datasets that have similar characteristics, such as achievement status data of unmet need targets in East Java, Indonesia and percentage of population poor in Indonesia. The results showed that the truncated spline nonparametric regression model performed better than the binary logistic regression model. For future research, it is recommended to test this model on datasets with more complex and varied predictor variables. In addition, this model can serve as a basis for developing parameter significance tests, which allow for a more in-depth evaluation of the developed model.

## Limitations

1. The regression model used is the linear Truncated Spline nonparametric regression model in the implementation of the achievement status data of unmet need target in East Java, Indonesia and percentage of poor population in Indonesia.
2. The method used in selecting the optimal knot point is the smallest AIC.
3. The number of optimal knot points used in the implementation of datasets are *u* = 1, 2, 3.
4. The estimation method used in the categorical data Truncated Spline nonparametric linear regression model is Maximum Likelihood Estimation (MLE).
5. The best model comparison method uses deviance value and classification criteria.
6. The data used is secondary data from the publication of BKKBN and BPS.

## Ethics statements

The data we use in this research is secondary data that we collected from publication of the National Population and Family Planning Agency (BKKBN) and Indonesian Central Bureau of Statistics (BPS). The data is available.

## Supplementary material and/or additional information [Optional]

None

## CRediT authorship contribution statement

**Afiqah Saffa Suriaslan:** Conceptualization, Methodology, Software, Writing – original draft, Visualization. **I Nyoman Budiantara:** Conceptualization, Methodology, Writing – review & editing, Validation, Supervision. **Vita Ratnasari:** Conceptualization, Methodology, Writing – review & editing, Validation, Supervision.

## Declaration of competing interest

The authors declare that they have no known competing financial interests or personal relationships that could have appeared to influence the work reported in this paper.

## Data Availability

data was used for research described in the article
